# Supplementary figures and images for: Association between Prescribing and Intoxication Rates for Selected Psychotropic Drugs: A Longitudinal Observational Study
Source: Pharmaceuticals (Basel). 2024 Jan 22;17(1):143. doi: 10.3390/ph17010143 (PMC10818633; doi:10.3390/ph17010143)

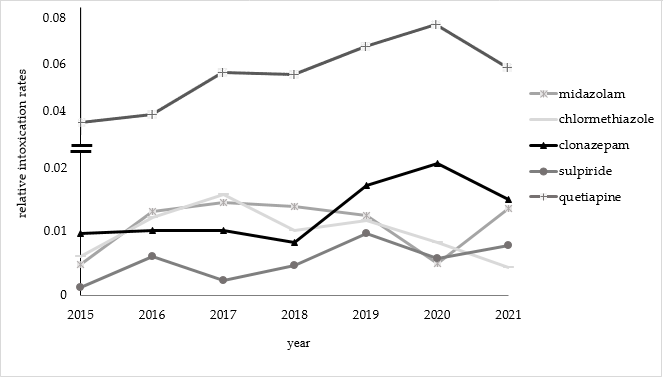

Supplement: Supplementary file 1 [file pharmaceuticals-17-00143-s001.zip › Supplementary Figure 1, Relative intoxication rates for selected psychotropic drugs, 2015-2021.PNG]

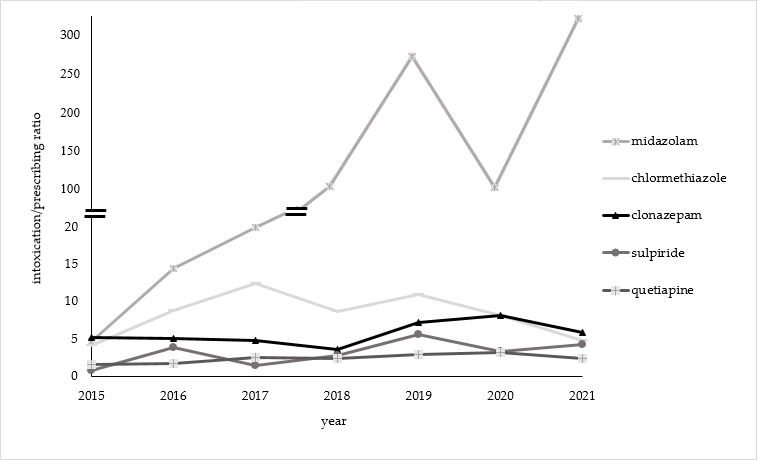

Supplement: Supplementary file 1 [file pharmaceuticals-17-00143-s001.zip › Supplementary Figure 2, Intoxication-prescribing ratio for selected psychotropic drugs, 2015-2021.PNG]
